# Revisiting Latex-Fruit Syndrome after 30 Years of Research: A Comprehensive Literature Review and Description of Two Cases

**DOI:** 10.3390/jcm13144222

**Published:** 2024-07-19

**Authors:** Weronika Gromek, Natalia Kołdej, Szymon Świtała, Emilia Majsiak, Marcin Kurowski

**Affiliations:** 1 Polish-Ukrainian Foundation of Medicine Development, Nałęczowska 14, 20-701 Lublin, Poland; weronikaa.gromek@gmail.com; 2 Department of Immunology and Allergy, Medical University of Lodz, 90-419 Lodz, Poland; natalia.koldej@student.umed.lodz.pl (N.K.); szymon.switala@student.umed.lodz.pl (S.Ś.); 3 Student Scientific Association for Allergy, Asthma, and Immunology at the Department of Immunology, Rheumatology, and Allergy Clinic, Medical University of Lodz, 90-419 Lodz, Poland; 4 Department of Health Promotion, Faculty of Health of Sciences, Medical University of Lublin, Staszica 4/6, 20-081 Lublin, Poland

**Keywords:** latex-fruit syndrome, latex allergy, fruit allergy, cross-reactivity

## Abstract

Thirty years have passed since the concept of latex-fruit syndrome (LFS) was first introduced. Since then, this phenomenon, characterized by cross-reactivity between natural latex rubber allergens and certain fruit allergens, has been extensively studied. This literature review sought to determine the prevalence of LFS in latex-allergic patients, identify the most common cross-reactions with fruit allergens in individuals with LFS, and understand the clinical manifestations of this syndrome. An extensive literature search was carried out using PubMed and Scopus databases, while applying the Preferred Reporting Items for Systematic Reviews and Meta-Analysis methodology. The analysis of original studies revealed a wide variation in LFS prevalence (4–88%) influenced by diverse diagnostic tools, different geographical regions, and the size of study populations. Our findings indicate that the most prevalent allergenic fruits in patients with LFS are banana, avocado, kiwifruit, and papaya. After evaluating the symptoms of the fruit hypersensitivity of patients with LFS, the clinical manifestation of hypersensitivity constituted 73% of systemic allergy symptoms and only 27% of reported symptoms described the localized allergy. Furthermore, the clinical picture of latex-fruit syndrome is illustrated through two cases, one typical and one with an unusual presentation. Their clinical features were assessed and contrasted utilizing different anaphylaxis severity grading criteria. To properly manage LFS, it is essential to establish standardized diagnostic criteria and severity grading systems, as these are crucial for accurate diagnosis and effective treatment.

## 1. Introduction

Thirty years have passed since Bianco et al. first established the link between a natural rubber latex (NRL) allergy and food hypersensitivity, known nowadays as latex-fruit syndrome (LFS). LFS was diagnosed basing on clinical history and positive skin prick testing. In their study of 25 patients with a latex allergy, features of LFS were present in 52% (*n* = 13). The symptoms of fruit hypersensitivity were predominantly triggered by avocado (*n* = 9), chestnut (*n* = 9), banana (*n* = 7), kiwi (*n* = 5), and papaya (*n* = 3) [1].

Over the last 30 years, the results of extensive research have been enhancing our understanding of LFS, uncovering its complexities. Patients with LFS may display a variety of severities of allergic reactions, from local symptoms such as oral allergy syndrome to systemic reactions like anaphylaxis [2]. Frequently, challenges arise with regard to the assessment of symptoms and the determination of the severity of allergic reactions to latex and cross-reacting allergens. Therefore, the proper defining, diagnosing, and severity assessment of anaphylaxis is of the utmost importance from the point of view of proper management as well as epidemiological research. There is an ongoing discussion regarding the utility of different systems of its grading and classification. Several proposals of the grading systems for generalized reactions have been proposed and are used concomitantly. Different criteria and different clinical characteristics are used for grading in each system, and these discrepancies may lead to considerable differences in the assessment.

The primary aim of this review was to present the prevalence of latex-fruit syndrome within the latex-allergic population and to pinpoint the specific fruits that elicit this syndrome. We present a thorough overview of the current knowledge surrounding latex-fruit syndrome and defining future perspectives. Additionally, we describe two cases of LFS, one typical and another with an unusual presentation, to present an overview of current concepts in LFS pathogenesis and the clinical picture. Having this in mind, and based on our cases’ description, we have compared the grading of the severity of anaphylactic reaction as a manifestation of latex-fruit syndrome assessed with the use of different anaphylaxis grading systems.

## 2. Natural Rubber Latex (NRL) Allergy

The implementation of disposable gloves in the early 1980s contributed to the increase in hygiene levels in the healthcare sector, but their widespread use led to more frequent latex exposure among sensitive individuals, causing possible problems for medical personnel and for patients [3,4]. In the medical setting, latex can be found, among others, in syringes, stethoscopes, tourniquets, dental dams, catheters, IV tubing, electrode pads, respirators, drainage tubes, and condoms. Currently, a warning must be displayed on most medical devices containing NRL. However, it has not always been properly performed in every country [3]. The prevalence of latex allergies is estimated to be around 4% in the general population [4]. The clinical presentations are primarily linked to IgE-mediated type I hypersensitivity reactions [3,5]. A latex allergy typically manifests as cutaneous symptoms, such as pruritus and urticaria, but in the most severe cases, anaphylaxis can occur. Additionally, a latex allergy can progress as type IV hypersensitivity which is T-cell-mediated and manifests as allergic contact dermatitis with symptoms typically developing within 24–48 h of exposure [5,6]. Nevertheless, those responses are often caused by additional substances incorporated during the manufacturing of latex such as accelerators or antioxidants (e.g., carbamates, thiurams) [7]. The evidence suggests that healthcare workers are at a higher risk of latex sensitization as compared with the general population [5]. Another important risk group for NRL allergies is individuals with spina bifida which is an inborn condition characterized by the incomplete development of the neural tube [8]. Given the need for surgical intervention in spina bifida cases [9], these individuals may experience prolonged exposure to latex-containing materials, further increasing the probability of sensitization [5]. Other groups of patients repeatedly exposed to latex include those undergoing surgical procedures, cesarean section, bladder exstrophy, as well as individuals subject to anesthesia or catheterization. The prevalence of latex allergies in healthcare professionals and vulnerable individuals is 9.7% and 7.2%, respectively [4]. Currently, in developed countries, the problem is gradually diminishing due to the development and commercialization of products with synthetic latex substitutes and to the increased social awareness [3].

## 3. Latex and Its Allergens

Natural latex rubber is obtained from a milky emulsion coming from *Hevea brasiliensis* (order Euphorbiales, family Euphorbiacees), a rubber tree which is commercially cultivated in Thailand, Malaysia, and India [7,10]. The synthesis of milky fluid is executed by laticifers—highly specialized plant cells that create a branched-out system of linear tubes inside the plant body [7,11]. The procedure of scarifying the trunk of *Hevea brasiliensis* is carried out during latex collection. To prevent the coagulation, an ammonia treatment is applied to the fresh milky emulsion [12]. Latex solution comprises water (55–65%), cis-1,4-polyisoprene rubber (34%), sugars (1.0–2.0%), sterol glycosides (0.1–0.5%), resins (1.5–3.5%), ash (0.5–1.0%), and proteins (2.0–3.0%) [10]. Proteins included in this emulsion play a vital role in the defense of *Hevea brasiliensis* against herbivores and pathogens. The fresh latex solution undergoes the process of ultra-centrifugation and leads to three layers (rubber phase, C-serum, and B-serum) [7].

Among 250 identified proteins in these three layers, there are 60 compounds that can bind specific human immunoglobulins E (sIgE) and 15 of them are described as allergens by the World Health Organization and International Union of Immunological Societies (WHO/IUIS) Allergen Nomenclature Sub-Committee. The aforementioned proteins’ names originate from the name of the latex tree—*Hevea brasiliensis*—and are assigned as Hev b with numbers ranging from 1 to 15 [13]. A comprehensive description of the molecules derived from *Hevea brasiliensis* is presented in Table 1. There are four proteins, Hev b 1, Hev b 3, Hev b 5, and Hev b 6.01, which are recognized as major allergens (responsible for allergic reactions among over 50% of individuals with an allergy to a specific allergen) among spina bifida patients, and Hev b 5 and Hev b 6.01 among healthcare professionals [3]. Molecules such as Hev b 1 (rubber elongation factor) and Hev b 3 (small rubber particle protein) are in the uppermost parts of the solution called the rubber phase. Hev b 5—an acidic structural protein—can be found in C-serum that makes up for the middle part of the solution. In this part, proteins that come from the cytosol of lacifer cells can be found. The last major allergen, Hev b 6.01, is at the bottom of the latex solution, in part known as B serum. In this part, proteins from specialized cell organelles known as “lutoids” can be found; among them, one can find pathogenesis-related proteins [7,10].

## 4. Definition of Latex-Fruit Syndrome

Latex-fruit syndrome is defined as a phenomenon characterized by cross-reactivity between NRL and plant-derived food allergens due to the similarity of the sequence homology of IgE-binding epitopes [42]. To achieve a more profound comprehension of LFS, multiple studies have aimed to determine responsible allergens. The development of component-resolved diagnostics (CRD) have played a vital part in this identification process. The main allergens of latex involved in LFS include Hev b 2 [16], Hev b 6.02 [22,43], Hev b 7 [44], Hev b 8 [45], and Hev b 12 [46]. Further research on the genetic basis of this phenomenon has shown that latex-fruit syndrome is linked to HLA-DQB1 *0201, DRB1 *0301, and *0901 and HLA-DR functional group E [47].

## 5. Diagnosis of LFS

The diagnosis of LFS syndrome is based on a thorough history, with particular attention to the reactivity to latex and potential cross-reactions [10]. An assessment of potential cross-reactivity and contributing factors should include:

- The symptoms of an allergy to fruit;
- The symptoms appearing in an occupational setting;
- The role of potential cofactors.

As the next diagnostic steps, the European Academy of Allergy and Clinical Immunology recommends employing subsequent tools, such as skin prick tests, serological tests for sIgE, and CRD, to gain insights into the patient’s sensitization and potential cross-reactivity [7].

## 6. Materials and Methods

**Figure 1:**
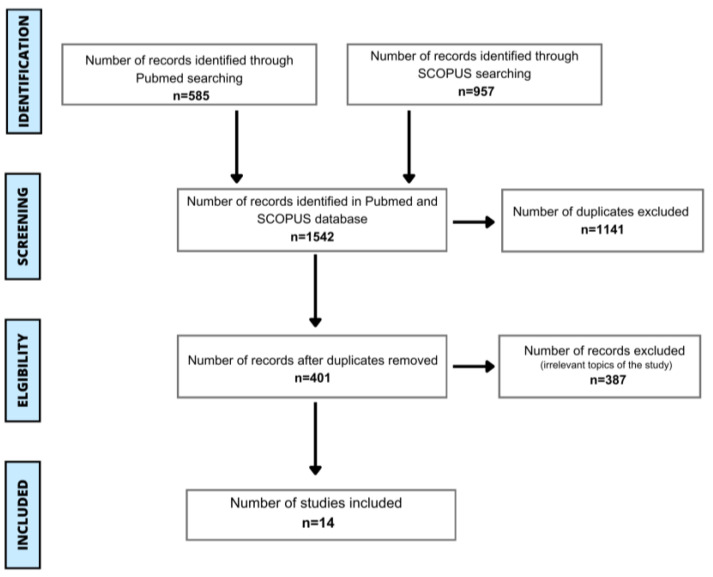
PRISMA flow chart displaying the process of sources’ selection.

In this literature review, we searched PubMed and Scopus databases to identify articles relevant to latex-fruit syndrome. Following search terms were applied: “latex-fruit AND syndrome”; “latex AND fruit AND syndrome”; “latex-fruit AND allergy”; and “latex AND fruit AND allergy”. The PRISMA guidelines were applied in this review, as shown in Figure 1 [48]. In the initial phase of screening, two independent authors evaluated the title and abstracts. Any conflicts were settled by either discussing them until a unanimous decision was reached or by seeking input from a supervisor. Subsequently, the articles were introduced into Excel to eliminate duplicates. After conducting deduplication, we analyzed and selected the records. A significant phase of the screening process was confirming that the subject of the articles met the objectives of this review and that the information presented was accessible in the English language for inclusion. Extracted data covered detailed information about epidemiology of latex-fruit syndrome, the most prevalent allergenic fruit in latex-fruit syndrome, tested allergens, and symptoms. Out of 401 screened articles, 387 were excluded because they were irrelevant to the subject of the research, or their full text was not accessible in English. The literature explored various aspects of the LFS, from which we gathered the necessary information to address our inquiries. Further assessment, involving statistical analysis and visualization, was executed using the Excel software (Microsoft^®^ Excel for Mac, version 16.87, Microsoft Corporation, Redmond, WA, USA).

Beyond providing the comprehensive clinical review, we evaluated the symptoms of 2 LFS patients. Two independent reviewers employed several anaphylaxis grading systems to assess the severity of allergic symptoms in patients with LFS, specifically focusing on the clinical manifestation of cross-reactions with fruits. By utilizing multiple assessment tools, we aimed to present a comprehensive and unbiased analysis of patients with LFS. Furthermore, to settle any disagreements in symptom classification, the supervisor (M.K.) was consulted.

## 7. Results

We have identified and analyzed 14 relevant articles originating from Austria, Belgium, Brazil, Canada, Finland, France, Germany, Italy, Taiwan, and the United States of America (USA), including 825 patients in total. Based on the available literature, our objective was to address the clinically relevant questions listed below:

1. What is the prevalence of latex-fruit syndrome?
2. What are the most prevalent cross-reactions with fruit allergens in subjects with latex-fruit syndrome?
3. What is the clinical manifestation of latex-fruit syndrome?

## 8. Epidemiology of LFS

**Figure 2:**
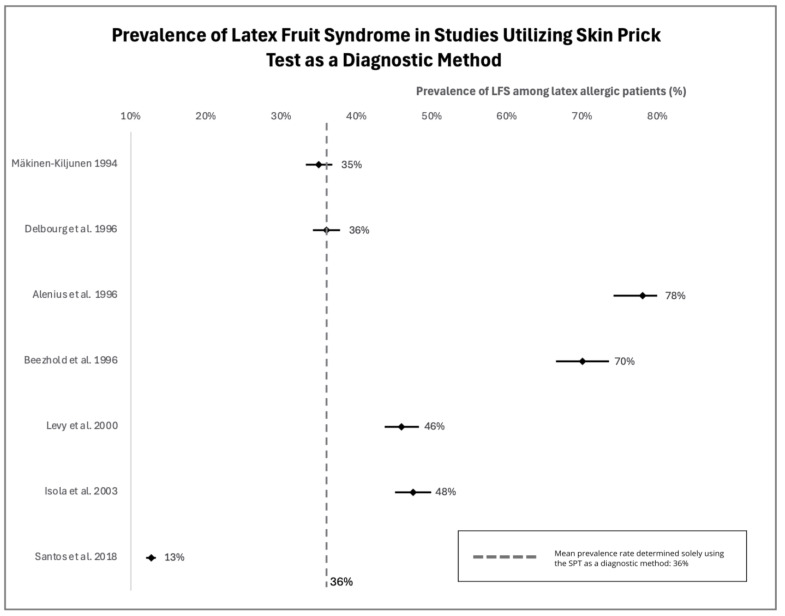
Forest plot of prevalence of latex-fruit syndrome in latex allergic patients based on skin prick test as a diagnostic method [52,53,56,57,58,60,61].

**Figure 3:**
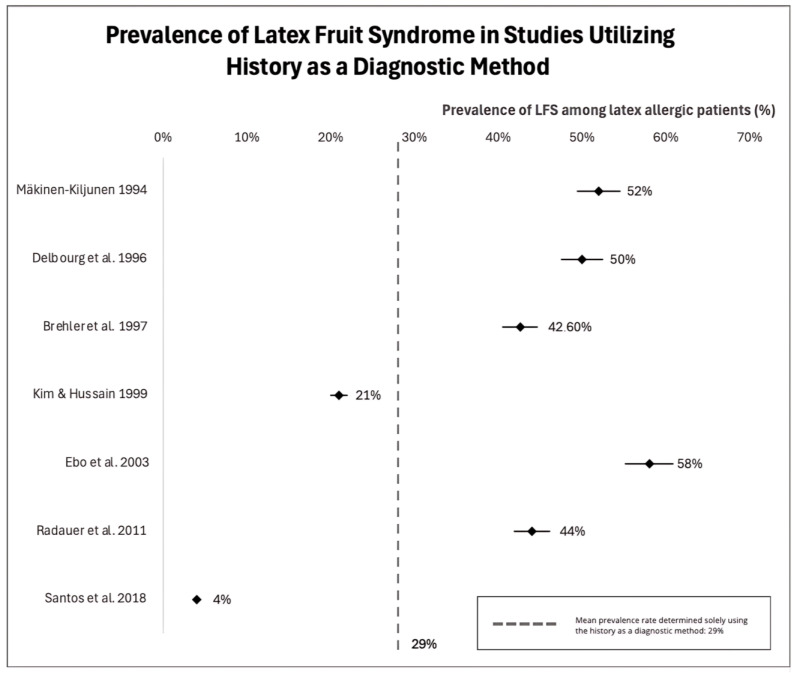
Forest plot of prevalence of latex-fruit syndrome in latex allergic patients based on clinical history as a diagnostic method [49,50,52,53,56,59,62].

Studies on the prevalence of LFS among latex-allergic individuals showed variable results. In these studies, researchers assessed LFS using variable methods: history and/or sIgE tests and/or SPT. The analysis of original studies revealed a wide variation in LFS prevalence, between 4% and 88%, which was influenced by the use of diverse diagnostic tools, different geographical regions, and the size of study populations. The highest prevalence of LFS (88%), basing on sIgE presence, was ascertained in a study from Belgium [49], while in another study of a similar design, LFS was described in 69.1% of German subjects [50]. Based on studies where a diagnosis was made based on SPT, the highest incidence of LFS (78%) was revealed in a study from Finland [51]. The lowest prevalence has been identified in Brazil—12.7% based on SPT [52], as presented in forest plots in Figure 2. The mean prevalence estimated based solely on SPT was equal to 36%. The prevalence of LFS is estimated between 4% and 58% when patients’ clinical history is considered and these data are presented in Figure 3 as a forest plot [49,52]. The mean LFS prevalence according to assessments based solely on the patient’s history has been estimated at 27%.

**Figure 4:**
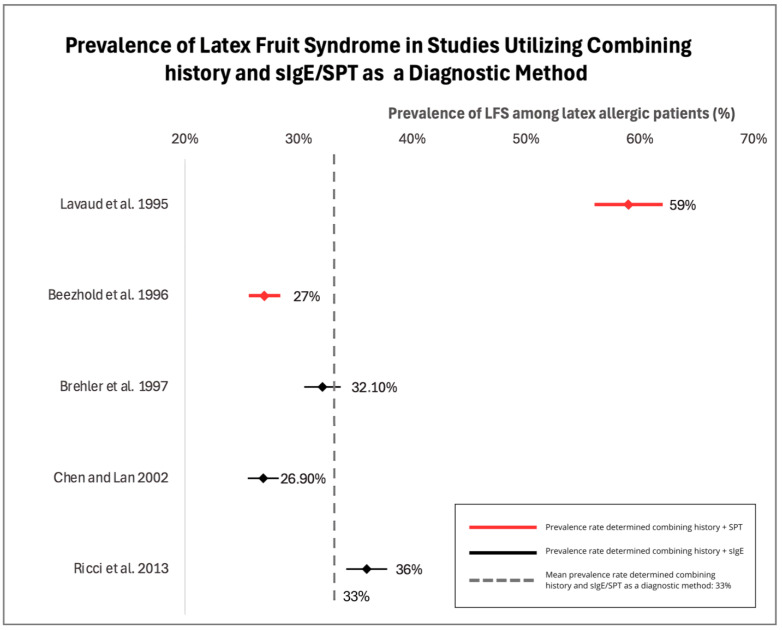
Forest plot of prevalence of latex-fruit syndrome in latex allergic patients using combined history and diagnostic methods (serum immunoglobulin E/Skin Prick Test) [2,50,54,55,58].

Such considerable variability mainly results from different diagnostic criteria in the assessment of fruit allergies in latex-allergic patients. Comparing patient history with the results of sIgE levels in serum seems to be the most precise method to assess LFS presence [53]. Upon the closer examination of the three studies which diagnosed LFS based on history and sIgE, the prevalence shows more precise results that range from 26.9 to 36% and these data are presented in Figure 4 as a forest plot [2,50,54]. The mean prevalence estimated based on the combined history and diagnostic methods (sIgE/SPT) was estimated to equal 33%. A summary of the research on the prevalence of LFS can be seen in Table 2.

**Table 2:** Summary of original studies on prevalence of latex-fruit syndrome assessed based on various diagnostic methods (clinical history and/or skin prick test and/or serum immunoglobulin E) found in Pubmed and Scopus prior to March 2024.

	Autor	Number of Participants	Country	Tested Allergens	The Most Prevalent Allergenic Fruit	Prevalence of LFS (Based on…)
1	Mäkinen-Kiljunen 1994 [53]	n = 47	Finland	Latex, Banana	Banana	52% (History) 35% (SPT)
2	Lavaud et al. 1995 [55]	n = 17	France	Latex, Banana, Avocado	Avocado	59% (History + SPT)
3	Delbourg et al. 1996 [56]	n = 19	France	Latex, Banana	Banana	50% (History) 36% (SPT)
4	Alenius et al. 1996 [57]	n = 22	Finland	Latex, Banana	Banana	45% (Immunobloting) 78% (SPT)
5	Beezhold et al. 1996 [58]	n = 47	Canada	Avocado, Potato, Banana, Tomato, Chestnut, Kiwi, Pineapple, Milk	Avocado	27% (History + SPT) 70% (SPT)
6	Brehler et al. 1997 [50]	n = 136	Germany	Latex, Papaya, Papain, Avocado, Chestnut, Banana, Ficus Spp., Passion Fruit, Melon, Mango, Kiwi, Peach, Pineapple, Tomato, Guava	Banana and kiwi	42.6% (History) 69.1% (sIgE) 32.1% (History + sIgE)
7	Kim and Hussain 1999 [59]	n = 137	USA	Latex, Banana, Avocado, Kiwi, Tomato, Watermelon, Peach, Carrot, Apple, Chestnut, Cherry, Coconut, Apricot, Strawberry, Loquat	Banana	21% (History)
8	Levy et al. 2000 [60]	n = 24	France	Avocado, Banana, Apple, Peach Celery, Kiwi, Mango, Tomato, Chestnut, Cantaloupe, Pineapple, Papaya	Banana	46% (SPT)
		n = 20			Papaya	24% (SPT)
9	Chen and Lan 2002 [2]	n = 26	Taiwan	Latex, Avocado, Apple, Pear, Kiwi, Papaya, Pineapple, Peach, Cherry, Plum, Apricot, Banana, Melon, Nectarine, Grape, Fig, Passion Fruit, Tomatoes, Celery, Carrot, Hazelnut, Chestnut, Potatoes	Unknown	26.9% (History + IgE)
10	Ebo et al. 2003 [49]	n = 42	Belgium	Avocado, Banana, Chestnut, Fig, Kiwi, Papaya, Peanut, Pineapple, Tomato, Ficus Benjamina	Papaya (sIgE) Banana (history)	88% (sIgE) 58% (History)
11	Isola et al. 2003 [61]	n = 82	Italy	Kiwi, Banana, Avocado, Papaya	Kiwi	47.5% (Skin test)
12	Radauer et al. 2011 [62]	n = 34	Austria	Banana, Avocado	Banana	44% (History)
13	Ricci et al. 2013 [54]	n = 22	Italy	Latex, Kiwi, Chestnut, Peach, Cherry, Apple, Melon	Kiwi	36% (History + sIgE)
14	Santos et al. 2018 [52]	n = 150	Brazil	Banana, Latex	Banana	12.7% (SPT) 4% (History)

LFS, latex-fruit syndrome; SPT, skin prick test; sIgE, specific human immunoglobulins E; n, number.

## 9. The Most Prevalent Allergenic Fruit in Individuals with LFS

One of the first systematic reviews conducted by Blanco and colleagues in 2003 showed that the fruits most frequently reported as responsible for the occurrence of latex-fruit syndrome were banana, avocado, kiwifruit, and chestnut [42]. In our analysis based on 14 different studies, the most frequently reported foods were banana, avocado, kiwi, and papaya. Banana has been reported as the most common LFS-inducing fruit in nine studies from seven countries: Austria, Belgium, Brazil, France, Finland, Germany, and the USA [49,50,52,53,56,57,59,60,62]. Avocado has been identified as the most frequent LFS trigger in a study conducted in France [55] and in another study from Canada [58]. Kiwi has been identified as the most common LFS trigger in three studies from two countries: Germany and Italy [50,54,61]. Papaya has been identified as the most common LFS inducer in two studies from two countries: France and Belgium [49,60]. A summary of most prevalent causative fruits in individuals with LFS based on can be seen in Table 3. Additionally, individuals with LFS have been reported to experience allergic reactions to other foods, such as bell pepper, potato, pineapple, passion fruit, tomato, Ficus spp., mango, melon, peach, guava, jackfruit, cassava, turnip, and zucchini [42,63,64,65,66].

One of the allergen groups which can help explain the phenomenon of cross-reactivity of latex and fruit is chitinases [66,67]. Their biochemical role is to catalyze the hydrolysis of β-1,4-N-acetyl-D-glucosamine bonds in chitin polymers. This enzyme is universally present in nature. It is synthesized by bacteria, fungi, insects, plants, and vertebrates [66]. Proteins from the chitinase group can be found in bananas, avocados, and papayas. In bananas, the Mus a 2 protein and, in avocados, the Pers a 1 protein have been identified as allergens belonging to the class I chitinases. They may show cross-reactivity with class I chitinases from latex: Hev b 6.01 (Prohevein) and Hev b 6.02 (Hevein). Additionally, chitinases have been identified in chestnut (class I), tomato (class II), Indian jujube fruit (class III), raspberry (class III), and grapes (class IV). In the case of kiwi and papaya, scientists have yet to identify an allergen with this exact structure and biochemical activity [67]. Other latex allergens responsible for cross-reactions with fruits may belong to profilins, glucanases, or non-specific lipid transfer proteins (nsLTPs) [3].

## 10. Clinical Picture of LFS

To address the issue of the clinical manifestation of LFS, out of 14 previously identified studies including a total number of 825 patients with latex allergy and/or LFS, 7 papers which contained descriptions of symptoms of fruit hypersensitivity were selected for further analysis. These selected papers described, in total, 353 patients with a latex allergy who originated from six countries: Austria, Belgium, Finland, France, Italy, and the USA. In this group, we have identified 172 patients with latex-fruit syndrome [49,55,56,57,59,61,62]. After evaluating 269 symptoms of fruit hypersensitivity of patients with LFS, we have noticed 72 reports (27%) of localized allergies such as oral allergy syndrome (*n* = 39), itchy mouth (*n* = 21), contact urticaria (*n* = 9), glossitis (*n* = 2), and lip pruritus (*n* = 1). In the process of the evaluation of systemic allergy reactions to fruits among latex-allergic patients, we have analyzed 197 reported systemic symptoms (73%) and categorized them into organ-related symptoms based on the World Allergy Organization (WAO) grading system for systemic reactions, upgraded in 2024 [68]. The systemic symptoms categorized based on the WAO 2024 grading system are summarized in Table 4. The mucocutaneous symptoms were reported 109 times, among these, the most prevalent single systemic symptoms were urticaria *(n* = 55) and angioedema (*n* = 33). Asthma symptoms were reported 38 times. Additionally, anaphylaxis occurred 14 times. Other symptoms which were less commonly reported were gastrointestinal disorders, rhinoconjunctivitis, rhinitis, eczema, facial edema, atopic dermatitis, generalized urticaria, pruritus, and erythema.

## 11. Case Reports

**Patient #1**

**Patient history:** A 33-year-old female was referred to the allergist with the purpose of a diagnostic evaluation of symptoms including generalized urticaria without angioedema as well as vomiting after banana consumption. In the past, she reported a generalized urticarial rash and pruritus, as well as episodes of ocular edema and dyspnea immediately following the consumption of either banana or chives. The symptoms were of mild intensity, subsided promptly, and did not make the patient seek medical advice. Additionally, transient tongue stiffness occurred once after kiwi fruit consumption. The patient recalls having eaten at that time other foods that she consumes daily without experiencing any allergic symptoms.

Perennial rhinitis symptoms, mainly nasal obstruction, with seasonal exacerbations in March, April, and May, have been present for several years. No allergy diagnostic work-up has been performed so far and the patient has been using over-the-counter oral cetirizine and decongestant nasal drops on an on-demand basis. She claims to have a poor tolerance of antihistamines (somnolence) and thus relies more frequently on oral calcium preparations.

The patient’s additional medical history includes uterine fibroids and thyroidectomy due to nodular goiter. Her current permanent pharmacotherapy includes oral levothyroxine 125 µg QD. The patient is an office employee, uses e-cigarettes daily, and lives with a dog. No family history of allergies or hypersensitivity has been reported.

Clinical presentation: Symptoms developed within minutes after eating a banana and the patient presented herself at the emergency department of the local hospital. No discharge report could be retrieved; however, the patient denies loss of consciousness, blood pressure drop, and any other symptoms other than those observed initially. She was given IV treatment, most probably an antihistamine and a steroid, which could not be confirmed due to the patient’s failing to retrieve any treatment documentation.

During the consultation 4 weeks later, the patient reported no skin symptoms, and no cutaneous signs were ascertained either. Impaired nose patency with moderate inferior turbinate swelling was noted on an anterior rhinoscopy with a moderate subjective feeling of nasal obstruction. No other upper or lower airways abnormalities could be detected upon physical examination.

**Initial diagnosis:** allergic rhinitis; polyvalent fruit allergy, possible cross-sensitization with latex allergens.

Diagnostic procedures: Skin prick tests were performed with a standard panel of seasonal and perennial airborne allergens, including house dust mites, pollen (alder, hazel, birch, grasses, rye, and mugwort), mold spores (Alternaria, Cladosporium) and cat and dog dander and, additionally, latex, banana, and kiwi extracts (Allergopharma, Reinbek, Germany). The results were positive for kiwi and banana extracts. Allergen-specific immunoglobulins E (sIgE) were measured in serum using a Polycheck^®^ 30-allergen inhalation panel (Biocheck GmbH, Münster, Germany). Positive results were seen for birch pollen (0.56 kU/L) and dog dander (0.46 kU/L) (specific IgE for latex, banana, and kiwi extracts were assessed using ImmunoCAP (Pharmacia Diagnostics, Uppsala, Sweden), revealing 1.18 kU/L for banana, 1.96 kU/L for kiwi, and 0.50 kU/L for latex-specific IgE). Component-resolved diagnostics were not performed due to a lack of reimbursement of the procedure in the public healthcare system and financial limitations on the patient’s side. A specific IgE concentration of 0.35 kU/L was considered as a threshold value for positive testing. Based on the patient’s symptoms and results of the laboratory tests, the diagnosis of persistent allergic rhinitis with sensitization to birch pollen and dog dander as well as a diagnosis of a food allergy presenting as latex-fruit cross-reactivity was confirmed.

**Treatment:** Intranasal glucocorticosteroid (fluticasone furoate) at a daily dose of 110 µg and oral rupatadine 10 mg were commenced on a regular basis. Prescribed emergency on-demand self-administration treatment included a single dose of 30 mg oral prednisone and epinephrine in an autoinjector (300 µg/0.3 mL). The patient was provided with comprehensive instructions on epinephrine administration. Detailed information about potential cross-reacting allergen sources was given and the avoidance of potential elicitors of allergic reactions was recommended. The patient has been followed on subsequent visits at her GP and the allergic rhinitis symptoms responded to the prescribed treatment. Two years and four months after the diagnosis (June 2024), she experienced an episode of generalized urticaria without other systemic symptoms, which had not subsided after oral rupatadine and prednisone and required IV treatment and the emergency department, responding to 8 mg dexamethasone and 2 mg clemastine. This episode was not associated with food consumption or any other identifiable causative factor. Further diagnostic work-up has been scheduled while regular and on-demand treatment recommendations have been sustained.

**Patient #2**

**Patient history:** A 45-year-old female was referred to an allergist with the purpose of the identification of the eliciting factors of anaphylaxis she experienced after the consumption of a chestnut. In addition, the patient reported having experienced in the past such symptoms as localized edema of the lips and face during dental procedures associated with direct contact of oral mucosa with latex gloves or a rubber saliva ejector. In the latter case, limited lip swelling persisted for several hours after the procedure, but no throat or larynx swelling or other systemic symptoms have been reported before by this patient in relation to contact with latex products or food ingestion. In the past, she was also diagnosed with atopic dermatitis, which has been in remission for several years. However, the patient recalled having been suspected of a food allergy many years before. No medical records could be retrieved, yet she remembered that an elimination diet was recommended without conclusive results of the diagnostic procedures. Following those recommendations, the patient excluded poultry and carrots from her diet. She reported having reintroduced those foods shortly after, with no subsequent symptoms suggestive of an allergy or hypersensitivity.

Clinical presentation: Symptoms started appearing within 15 min after chestnut ingestion, with the rapid development of cardiovascular and neurological signs, including blood pressure falling to 60/40 and an abrupt loss of consciousness. The patient did not recall having experienced considerable pruritus, hives, or prodromal symptoms of anaphylaxis. The episode was treated with intramuscular epinephrine and the patient was hospitalized for 2 days. A diagnosis of anaphylactic shock was established in the hospital discharge report.

**Initial diagnosis:** food allergen-induced anaphylaxis, possibly within the frame of latex-fruit syndrome (chestnut–latex cross-reactivity)

Diagnostic procedures: Skin prick tests (Allergopharma, Reinbek, Germany) were performed with apple, banana, strawberry, peach, walnut, rye flour, wheat flour, cocoa, Dermatophagoides farinae, Dermatophagoides pteronyssinus, grasses / cereals, trees l, trees II, weeds, dog fur, cat fur, Alternaria alternata, Cladosporium, and histamine hydrochloride (10%) as the control solution. All yielded negative results which were serum-specific IgE for food allergens were assessed using the EUROLINE allergy diagnostic profile (Euroimmun Medizinische Labordiagnostika AG, Lübeck, Germany). Additionally, IgE specific for latex, hazelnut, celery, and cod allergens had been assessed using ImmunoCAP (Pharmacia Diagnostics, Uppsala, Sweden), depending on the ImmunoCAP testing availability at the time of consultation. A specific IgE concentration of 0.35 kU/L was considered as a threshold value for the test’s positivity. Due to the lack of commercially available sIgE tests for chestnut, the test was not performed. Component-resolved diagnostics were not performed due to a lack of reimbursement of the procedure in the public healthcare system and financial limitations on the patient’s side.

The concentration of latex sIgE was 3.24 kU/L which confirmed IgE-dependent latex sensitization. The diagnosis of latex-fruit syndrome was confirmed based on the patients’ symptoms following the exposure to chestnut and latex products, identifying the probable cross-reactivity between allergens from the two sources.

**Treatment:** The patient was prescribed intramuscular epinephrine in an autoinjector (300 µg/0.3 mL) and provided with comprehensive instructions on its administration. Moreover, a detailed account of possible cross-reactivity reactions between different fruits and latex allergens was presented to the patient. The avoidance of chestnut (identified as the suspected culprit food) and a self-assessment of possible symptoms associated with other foods’ ingestion were advised.

## 12. Differences between Grading Systems

We have presented here two cases of latex-fruit syndrome manifesting as severe allergic reactions. Table 5 shows a comparison of grades that attribute the severity of allergic reaction in both patients, using different grading systems. Discrepancies in grading contribute to the divergent classification of identical clinical history. For instance, patient 2 was classified as grade 3 according to the Ring [69] and Błażowski systems [70], whereas they were classified as a grade 4 according to Muller [71] and grade 5 according to Sampson, Cox, WAO (World Allergy Organization) 2024, and Dribin [68,72,73,74]. However, as it was shown in the case of patient 1, the grading of a generalized allergy according to different guidelines can also yield comparable outcomes. Subject 1 was categorized as grade 3 using all scales except Sampson [72], according to which the patient would be attributed grade 4 [68,69,72,73,74].

The ongoing debate on the most suitable grading system for allergic reactions continues, regardless of a long history of anaphylaxis grading. The differences between grading systems arise from diverse settings, populations, and reaction triggers, which were considered during development and validation. Furthermore, the scales vary in their recommended timing for administering epinephrine and classifying symptoms as anaphylaxis. The aftermath of a lack of consensus may induce confusion among clinicians regarding when to administer epinephrine in the event of anaphylaxis. Using one scale in comparison with another might contribute to a delay in the administration of epinephrine. In addition to that, the scales differ in the description of the symptoms. For instance, Dribin’s latest scale is the most detailed and accurate one so far, as it meticulously focuses on individual symptoms. Unfortunately, it might be difficult and may have to be applied by healthcare professionals. Whereas Mueller and Sampson scales seem to be easy to comprehend, they are less detailed though. Moreover, the scales differ in the number of grades they employ, which varies from 4 to 5. The scales created by Mueller, Ring, and Błażowski [69,70,71] include 4 grades, whereas the scales by Sampson, Cox, WAO 2024, and Dribin [68,72,73,74] include 5 grades of reaction. In addition, the scales do not include symptoms from every organ and system; for example, the Ring scale does not include neurological symptoms as elements of the clinical presentation of anaphylaxis [69].

Indications for epinephrine administration are not included in every grading system. Severity grading systems proposed by WAO [68] and Dribin et al. [74] state clearly that they have not been intended as treatment choice guidance. Contrarily, Cox et al. [73] and Błażowski et al. [70] recommend a certain intensity and presentation of anaphylaxis symptoms as the ones calling for epinephrine administration. In the case of the WAO grading system [68], indications for epinephrine administration are aligned with the clinical criteria of anaphylaxis, with provisions made for fast-developing generalized urticaria, which may require IM epinephrine, despite symptoms not attaining a sufficient grade of systemic intensity. Including clear and unequivocal recommendations on the necessity of epinephrine use into the anaphylaxis grading system, which is generally accepted and recognized worldwide, would certainly improve patient care and provide guidance for medical practitioners at all levels of patient management.

## 13. Limitations

Numerous limitations have been identified while conducting this literature review. Firstly, the heterogeneity in the diagnostic criteria and methodologies among the studies complicates the direct comparisons and the fusion of the findings. Researchers employed various diagnostics criteria to determine the prevalence of LFS, such as skin prick tests and sIgE measurements. The lack of standardized diagnostic criteria for latex-fruit syndrome across the studies means that what one study identifies as LFS might differ significantly from another study’s criteria. Researchers employed various diagnostic techniques to assess the prevalence, including skin prick tests and sIgE measurements. Researchers evaluated prevalence using diverse combinations of allergens, with some focusing on latex and banana, while others covered even up to 23 fruits. The diversity in sample sizes influences the universality of the conclusions. Studies with limited sample sizes may not present an accurate depiction of the prevalence of LFS, contributing to bias in the presented results.

Furthermore, the studies were conducted in diverse geographical settings, each characterized by unique dietary habits, environmental exposures, and genetic predispositions, which may affect the prevalence and manifestation of LFS. It is important to highlight that most of the studies have been performed in the regions with abundant access to a wide variety of tropical fruits that are affordable and widespread. These regions include affluent nations like Germany or France, as well as countries where the tropical fruits are cultivated, like Brazil. It is worth mentioning that a data absence is clear in various African and Asian nations, showing a need to bridge the gap in information on LFS within these regions, which are home to various tropical fruits. The current review’s results could be affected by these factors as well.

Finally, the time frame of 30 years of the research analyzed in the review can be considered as another constraint. Advancements in diagnostic techniques and the progress of environmental pollution and dietary habits throughout the years could impact the occurrence and comprehension of allergies and, therefore, LFS. As a result, former research may not accurately illustrate the present situation.

## 14. Future Perspectives

I. After three decades of thorough investigation into LFS, this syndrome presents a potential for additional investigation and comprehension. The continuous study and analysis of LFS have paved the way for plenty of opportunities for deeper exploration and insight into this field.
  → Given the heterogeneity in the prevalence rates documented in previous studies, it has become imperative to advocate for the implementation of epidemiological examinations that comprise a diverse range of populations. Future investigations should establish consistent and comparable diagnostic criteria while evaluating hypersensitivity to fruits among latex-allergic patients. These investigations ought to encompass examinations for a spectrum of allergens that can potentially induce hypersensitivity reactions. The wide range of available fruit allergens allows for the selection of individual allergens for testing by SPT and serum sIgE measurement. This allows for a more efficient diagnostic management of LFS.
  → Expanding the diagnosis with relevant allergenic components of fruits in the diagnostic process can play a crucial role in pinpointing the specific molecules responsible for LFS and exposing potential cross-reactive patterns. Such an approach can not only reinforce the diagnostic accuracy, but also help with creating therapeutic strategies tailored to an individual patient’s needs.
  → Raising awareness among healthcare professionals about the connection between a latex allergy and fruit hypersensitivity is vital for proper diagnosis and subsequent treatment. It is essential to increase the awareness of the healthcare professionals about the allergen components involved in LFS, facilitating an accurate diagnosis and appropriate treatment strategies.
  → In addition, future research that examines the specific characteristics of allergen molecules and, in the future, on epitopes involved in LFS should provide a more profound understanding of this syndrome and support the development of better diagnostic tools and treatment options.
  → Improving the warnings and labeling of latex items may aid in averting inadvertent contact and reducing the likelihood of allergic responses in individuals with LFS. Consequently, the implementation of a singular, globally recognized warning icon on products that include latex is worth considering.
  → Enhancing the accessibility of latex alternatives such as polyurethane and polyisoprene plays a pivotal role in diminishing the likelihood of allergic responses within the demographic of both healthcare practitioners and individuals who frequently encounter latex-based items. By implementing these substitutes, the healthcare industry could effectively mitigate the risks posed by latex allergies, thereby ensuring a conducive and risk-free environment for both medical professionals and patients alike.

II. Furthermore, addressing significant gaps in systemic allergy grading systems is imperative for standardizing clinical assessment and management practices.
  → Implementing a standardized tool for assessing generalized allergic reactions in hospital Emergency Departments (EDs) and allergology units is crucial for enhancing communication and ensuring prompt interventions. Moreover, developing a globally adaptable anaphylaxis reporting form based on established grading systems should enable consistent documentation and robust data collection for epidemiological analysis and quality improvement efforts in Hospital EDs.
  → The primacy of epinephrine in anaphylaxis management should be kept in mind by medical personnel, regardless of the grading scale. To achieve the best patient outcomes in LFS and related allergies, it is vital to ensure familiarity with epinephrine administration techniques and prioritize ongoing education and training initiatives.

## Figures and Tables

**Table 1:** Summary of latex allergens and their basic properties.

Molecule	Biochemical Name	Clinical Relevance	Cross-Reactivity	Route of Exposure	Reference
Hev b 1	Rubber Elongation Factor (REF)	Major in children with spina bifida	Papain	Contact	[3,14,15]
Hev b 2	Beta 1-3-glucanase	Minor	-	Contact	[16]
Hev b 3	Small rubber particle protein (SRPP)	Major in children with spina bifida	-	Contact	[3,17]
Hev b 4	Lecithinase homologue	Minor	-	Contact	[18,19]
Hev b 5	Acidic structural protein	Major in healthcare professionals	Kiwi	Contact	[20,21]
Hev b 6.01	Prohevein	Major healthcare workers and spina bifida	Banana, avocado	Contact	[3,20]
Hev b 6.02	Hevein	-	Banana, avocado, chestnut	Contact	[22,23]
Hev b 6.03	Hevein C	-		Contact	[24]
Hev b 7	Patatin-like protein	Minor	Solanaceae (tomato and potato)	Contact	[3,25,26]
Hev b 8	Profilin	Minor	Birch pollen, Ambrosia artemisiifolia, Capsicum annuum, Chenopodium album, and other allergenic sources containing profilins	Contact	[27,28]
Hev b 9	Enolase	Minor	Cladosporium herbarum and Alternaria alternata	Contact	[29,30]
Hev b 10	Manganese superoxide dismutase (MnSOD)	Minor	Aspergillus fumigatus	Contact	[31,32]
Hev b 11	Chitinase Class I	Minor	Ficus benjamina, banana, avocado, chestnut, wheat, kiwi, and other allergenic sources containing chitinases	Contact	[10,33,34,35]
Hev b 12	Non-specific lipid transfer protein type 1 (nsLTP1)	Minor	Apple, peach, bell pepper, banana, potato, avocado, and other allergenic sources containing nsLTPs	Contact	[7,36,37]
Hev b 13	Esterase	Major		Contact	[21,38,39]
Hev b 14	Hevamine	Minor		Contact	[40]
Hev b 15	Serine protease inhibitor	Minor		Contact	[41]

**Table 3:** Most prevalent causative fruits in individuals with LFS based on 14 studies published prior to March 2024.

Position in Ranking	Fruit	Number of Studies	Number of Countries	Reference
1	Banana	9	7	[49,50,52,53,56,57,59,60,62]
2	Kiwi	3	2	[50,54,61]
3	Avocado	2	2	[55,58]
	Papaya	2	2	[49,60]

**Table 4:** A summary of systemic symptoms of LFS based on the literature on prevalence of LFS found by March 2024.

Organ	Symptoms	Number
Mucocutaneus		109
	Urticaria	55
	Angioedema	33
	Eczema	7
	Facial edema	6
	Atopic dermatitis	3
	Generalized urticaria	2
	Edema	1
	Pruritus	1
	Erythema	1
Respiratory		57
	Asthma	38
	Rhinoconjunctivitis	12
	Rhinitis	7
Gastrointestinal		14
	Gastrointestinal	14
Cardiovascular		14
	Anaphylaxis	14
Other		3
	Eye syndrome	3

**Table 5:** A comparison of grades attributed to the severity of reaction in both patients with employment of different grading systems [68,69,70,71,72,73,74]. Bold red script indicates symptoms and their severity that require epinephrine administration according to a given grading system, whenever such requirement is stated explicitly. Epinephrine administration requirements shown only in patient #1. Clinical picture in patient #2 unequivocally requires epinephrine administration irrespective of the grading system. See table captions below for additional comments.

Symptoms	Grade Assigned in Accordance with a Given Severity Scoring System						
	Mueller [71]	Ring [69]	Sampson [72]	Cox [73]	Błażowski [70]	Dribin [74] ^$^	WAO [68]
**Case 1**							
Generalized pruritus	Grade 1	Grade 1	Grade 2	Grade 2 *	Grade 1	Grade 2 ^‡^	Grade 2 ^#^
Generalized urticaria	Grade 1	Grade 1	Grade 2	Grade 2 *	Grade 1	Grade 2 ^‡^	Grade 2 ^#^
Localized ocular angioedema	Not included	Grade 1	Grade 1	Grade 2 *	Grade 1	Grade 2 ^‡^	Grade 1
Tongue stiffness and swelling	Not included	Grade 1	Grade 1	Grade 2 *	**Grade 1 ** ^†^	Grade 2 ^‡^	Grade 1
Vomiting	Grade 2	Grade 2	Grade 2	Grade 3	**Grade 2**	Grade 2 ^‡^	Grade 2
Dyspnea	Grade 3	Grade 3	**Grade 4**	Grade 3	**Grade 2**	Grade 3	**Grade 3**
**Case 2**							
Blood pressure fall	Grade 4	Grade 3 **	Grade 5	Grade 5	Grade 3	Grade 5	Grade 4
Loss of consciousness	Grade 4	Grade 3 **	Grade 5	Grade 5	Grade 4	Grade 5	Grade 4

* As per grading system proposed by Cox et al. [73], presence of ≥2 symptoms indicated in Grade 1 criteria classifies the systemic reaction as Grade 2. ^‡^ As per grading system proposed by Dribin et al. [74], classifying the reaction as Grade 2 is justified by presence of any moderate skin symptom (in this case, generalized urticaria) or ≥2 mild symptoms (in this case, exemplified by an episode of emesis, and localized facial/mucosal swelling). ^†^ Epinephrine recommended if >1 system with Grade 1 symptoms involved. ^#^ WAO grading system (2024) has not been intended as guidance for treatment administration. Epinephrine administration should be imminent if WAO criteria for anaphylaxis are fulfilled; however, the authors of the WAO grading system recommend considering epinephrine injection in case of, among other, progressing generalized urticaria, as was the case in patient no. 1. NOTE: this recommendation has been intended for generalized progressing urticaria following SCIT injection. **^$^** Grading system proposed by Dribin et al. has not been intended as the guideline for management decisions. The authors indicate that reaction of any severity or combination of symptoms from different organs or systems may require epinephrine administration. ** According to the grading system proposed by Ring, symptoms in patient no. 2 are not listed as such, but fall within the definition of the current shock reaction.

## Data Availability

Not applicable.
